# Individual-based Modeling of Genome Evolution in Haplodiploid Organisms

**DOI:** 10.1093/gbe/evac062

**Published:** 2022-05-02

**Authors:** Rodrigo Pracana, Richard Burns, Robert L. Hammond, Benjamin C. Haller, Yannick Wurm

**Affiliations:** Organismal Biology Department, Queen Mary University of London, London, United Kingdom; Organismal Biology Department, Queen Mary University of London, London, United Kingdom; Department of Genetics and Genome Biology, University of Leicester, Leicester, United Kingdom; Department of Computational Biology, Cornell University, Ithaca, NY, USA; Organismal Biology Department, Queen Mary University of London, London, United Kingdom; Alan Turing Institute, London, United Kingdom

**Keywords:** Hymenoptera, ants, bees, selection efficacy, haploid males, fate of mutations, interpreting genome scans, arrhenotoky

## Abstract

Ants, bees, wasps, bark beetles, and other species have haploid males and diploid females. Although such haplodiploid species play key ecological roles and are threatened by environmental changes, no general framework exists for simulating their genetic evolution. Here, we use the SLiM simulation environment to build a novel model for individual-based forward simulation of genetic evolution in haplodiploids. We compare the fates of adaptive and deleterious mutations and find that selection on recessive mutations is more effective in haplodiploids than in diploids. Our open-source model will foster an understanding of the evolution of sociality and how ecologically important haplodiploid species may respond to changing environments.

**Significance**

In most species of ants, bees, and wasps, males carry a single copy of each chromosome. In consequence, the effects of natural selection on these species differ from the effects on the many other species in which males have two copies of each chromosome. However, it has not been easy to take this difference into consideration in genomic analysis. Here, we present a method to run computer simulations of genomes of such haplodiploid species. This will allow researchers to make accurate predictions of how bees, ants, and wasps evolve and will help in the interpretation of genomic datasets for these species.

Approximately 15% of all animal species, including ants, bees, wasps, thrips, and bark beetles, have a haplodiploid sex-determination system: haploid, unfertilized eggs develop into males, while diploid, fertilized eggs develop into females (Pamilo and Crozier 1981). These haplodiploid species display a huge diversity of morphologies and behaviors. Furthermore, solitary and social bees occupy essential ecological and agricultural roles as pollinators (Potts et al. 2016), and bees and ants are charismatic models for studying social evolution (Hölldobler and Wilson 1990).

We are only beginning to understand the molecular–genetic bases and constraints underpinning the evolution of haplodiploid species. A key challenge for interpreting genomic data sets from such species is that haplodiploid populations evolve differently from populations of purely diploid individuals. This is because recessive mutations are under the full effect of selection in haploid males but masked from selection in heterozygous diploid males. Such a fundamental difference in how selection works is likely to have important evolutionary consequences, as suggested by analytical models of allelic evolution in simple haplodiploid populations (Avery 1984; Charlesworth et al. 1987; Hedrick and Parker 1997). An important limitation of these models is that they lack the flexibility to consider complex demography and realistic genomic processes including genetic linkage. A solution to this is to use simulations, which have become an essential tool for the theoretical study of evolution and the interpretation of empirical genomic data (Bank et al. 2014). Simulations are used to generate null distributions of common measures of genetic diversity and selection (Voight et al. 2006; Bank et al. 2014; Ferrer-Admetlla et al. 2014), to compare competing hypotheses about the demographic and evolutionary histories of populations (Excoffier et al. 2013; Enard et al. 2014; Adrion et al. 2020), and to predict the effects of environmental changes on different species (DeAngelis and Mooij 2005; Owens and Samuk 2020). The absence of a simulation framework incorporating haplodiploidy has limited the exploration of genomic models of social evolution and the accurate interpretation of population genomic data for bees, ants, and other important species (Favreau et al. 2018; López-Osorio and Wurm 2020; Colgan et al. 2022).

To enable the simulation of genome evolution in haplodiploid organisms, we present an individual-based model built upon the SLiM software framework for forward evolutionary simulations (version 3.7; Haller and Messer 2019). To simulate haplodiploidy, we restricted male individuals to inherit one recombined genome from the female parent and possess an empty “null” second genome. We assigned a relative fitness of 1 + *s* to male carriers of mutations with selection coefficient *s*. The Supplementary Text includes more details. Our model can be extended to consider variation in recombination rates, selection, and complex demographic structures.

We tested whether our simulations follow the expectations from analytical models (Avery 1984; Charlesworth et al. 1987) by comparing the fates of mutations between haplodiploid and diploid populations. We first modeled neutral mutations (*s* = 0) in both types of population. As expected from evolution by drift, with a mutation rate of 10^−8^ on a genome of 10^6^ loci, 0.01 mutations were fixed per generation in both population types (fig. 1*A*). Because recessive mutations are fully exposed to selection in haploid males but masked in heterozygous diploid males, we subsequently tested whether selection on recessive mutations is more effective in haplodiploid populations (Avery 1984). This was indeed the case: advantageous recessive mutations (*s* > 0) fixed at a higher rate in haplodiploid populations, with stronger effects in simulations with larger selection coefficients (fig. 1*B*). Similarly, weakly deleterious recessive mutations (−0.001 ≤ *s* < 0) fixed at a lower rate in haplodiploid populations (fig. 1*C*). For more strongly deleterious mutations (*s* = −0.003 and *s* = −0.01), very few fixed in either type of population, likely because selection against them overpowered drift (Agrawal and Whitlock 2011; Huber et al. 2018).

**Fig. 1.**
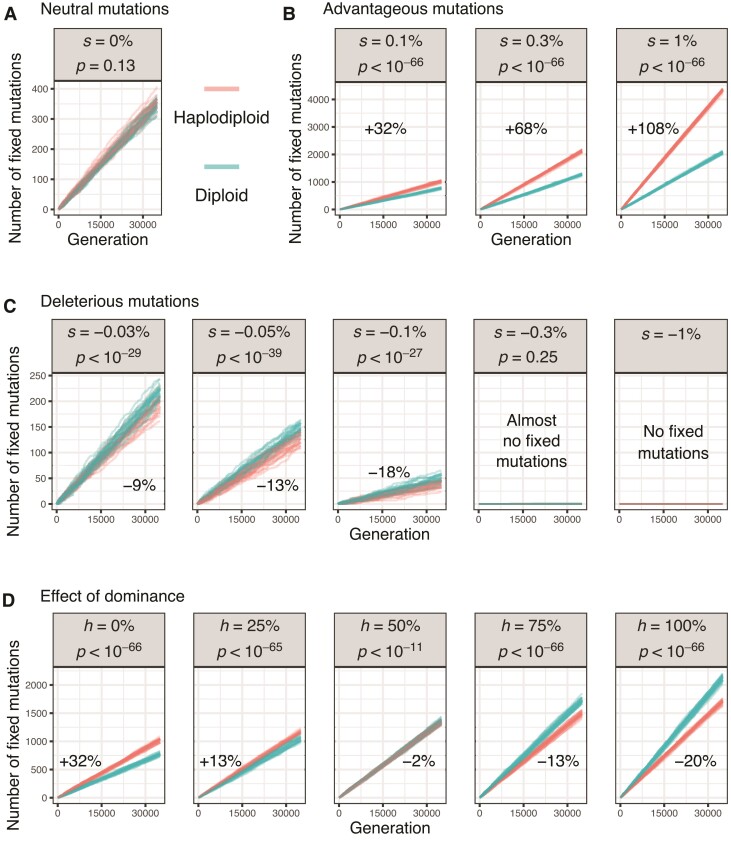
The effect of haplodiploidy on the fixation rate of (*A*) neutral mutations, (*B*) advantageous mutations, (*C*) deleterious mutations, and (*D*) advantageous mutations with different levels of dominance. Each line represents one simulation run (only 20 of 200 shown for each treatment). On each plot, we also show the average difference in the number of fixed mutations between haplodiploid and diploid simulations after 35,000 generations and a burn-in period of 15,000 generations (Wilcoxon rank-sum test; supplementary table S1, Supplementary Material online). For the simulations in (A) to (C), mutations were fully recessive (dominance coefficient *h* = 0) and had a range of selection coefficients (*s*, as shown); for the simulations in (D), mutations had *s* = 0.001 and a range of dominance coefficients (*h,* as shown). In (C), <5% of simulation runs with *s* = −0.3% and no simulation run with *s* = −1% had any fixed mutation.

In reality, most mutations are neither fully recessive (dominance coefficient *h* = 0, as considered in the simulations described so far) nor fully dominant (*h* = 1) (Orr 2010; Agrawal and Whitlock 2011; Huber et al. 2018). We therefore also compared fixation rates of advantageous mutations between population types across a range of dominance coefficients. For simulations of recessive mutations (*h* < 0.5), a greater number of advantageous mutations were fixed in haplodiploid populations than in diploid populations (fig. 1*D*). However, when mutations were dominant (*h* > 0.5), this pattern was reversed (fig. 1*D*). This somewhat counterintuitive reversed pattern likely occurs because haplodiploid populations have fewer chromosomes than diploid populations with the same number of individuals (1.5*N* vs. 2*N*, for a 1:1 sex ratio). Consequently, fewer mutations enter haplodiploid populations than diploid populations in each generation (1.5*Nμ* vs. 2*Nμ*), and thus, fewer mutations can ultimately fix (Charlesworth et al. 1987, 2018). Selection is still expected to be more effective in haplodiploid individuals for any given mutation with *h* < 1. Indeed, simulations where both population types have identical numbers of *chromosomes* rather than *individuals* showed a greater fixation rate for haplodiploid populations, with the magnitude of difference increasing as *h* decreases (supplementary fig. S1, Supplementary Material online).

The ability to simulate the evolution of haplodiploid genomes has the potential to fill major gaps in the study of the demographic and selective processes that have shaped the evolution of haplodiploid species. To date, considerable effort has been made to understand the impacts of haplodiploid reproduction on social evolution, particularly the asymmetry in within-family relatedness inherent to these species and the fact that females can control the sex of their offspring (Meunier et al. 2008). The model presented here will allow us to extend our understanding of the effects of haplodiploidy, for instance through the exploration of the capacity of haplodiploid species to resolve antagonistic selection between sexes or among colony members under different sex ratios (Vicoso and Charlesworth 2009; Eyer et al. 2019). Furthermore, the model will make it possible to test the hypotheses that higher efficacy of selection against recessive deleterious mutations in haplodiploid species may have facilitated the evolution of long lifespans in ant and bee queens and that such higher efficacy of selection may also reduce the degeneration of supergene regions of suppressed recombination (Stolle et al. 2019; Martínez-Ruiz et al. 2020). Our model should help us explore evolutionary processes in haplodiploid species and better understand how interactions between selection efficacy, population size, and migration can affect the ability of haplodiploid species to adapt to environmental change (Potts et al. 2016).

## Material and Methods

To be able to control reproduction rules explicitly in SLiM, we implemented our simulations of haplodiploid evolution as a non-Wright–Fisher (nonWF) model using SLiM v3.7. This version facilitates modeling of haploids by extending SLiM’s concept of null genomes to haploidy, and by adding a “haploidDominanceCoeff” property that controls fitness calculations in haploids versus diploids. The Supplementary Text provides modeling details. We compared this model, under different treatments, with a parallel nonWF model of diploid evolution. Because our model defines fitness in terms of reproduction success across non-overlapping generations, our nonWF model of diploid evolution is similar to a Wright–Fisher model (supplementary fig. S2, Supplementary Material online).

For each treatment, we simulated 200 populations of 1,000 males and 1,000 females, with genomes of 10^6^ loci, a mutation rate of 10^−8^, and a recombination rate of 10^−6^. The levels of dominance coefficient (*h*) and selection coefficient (*s*) used for each treatment are given in figure 1. In all cases, mutations had a haploid dominance coefficient of 1. Simulations ran for 35,000 generations after a burn-in of 15,000 generations. The burn-in period is important because haplodiploid and diploid populations with the same number of individuals have different effective population sizes and thus reach mutation–drift balance at different times (supplementary fig. S3, Supplementary Material online). Each simulation took ∼10 min; runtime would likely increase under more complex parameters (supplementary fig. S4, Supplementary Material online).

## Supplementary Material

Supplementary data are available at *Genome Biology and Evolution* online.

## Supplementary Material

evac062_Supplementary_Data

## Data Availability

Code, analysis scripts, and results of simulation runs are available at https://github.com/wurmlab/haplodiploid_simulations and https://wurmlab.com/data/simulating_haplodiploid_evolution.
