# Supplementary material for: Individual-based Modeling of Genome Evolution in Haplodiploid Organisms
Source: Genome Biol Evol. 2022 May 2;14(5):evac062. doi: 10.1093/gbe/evac062 (PMC9086950; doi:10.1093/gbe/evac062)
Supplement: evac062_Supplementary_Data [file evac062_supplementary_data.zip › haplodiploid_s_text.pdf]

Supplementary Text and Supplementary Figures for

Individual-based modeling of genome evolution in haplodiploid organisms

Rodrigo Pracana[1*§], Richard Burns[1*], Robert L. Hammond[2], Benjamin C. Haller[3], Yannick Wurm[1,4,§]

[1] Organismal Biology Department, Queen Mary University of London, London, UK

[2] Department of Genetics and Genome Biology, University of Leicester, Leicester, UK

[3] Department of Computational Biology, Cornell University, Ithaca, NY, USA

[4] Alan Turing Institute, London, UK

* Joint first authors

[§] Corresponding authors: rodrigopracana@gmail.com, y.wurm@qmul.ac.uk

Supplementary Figures

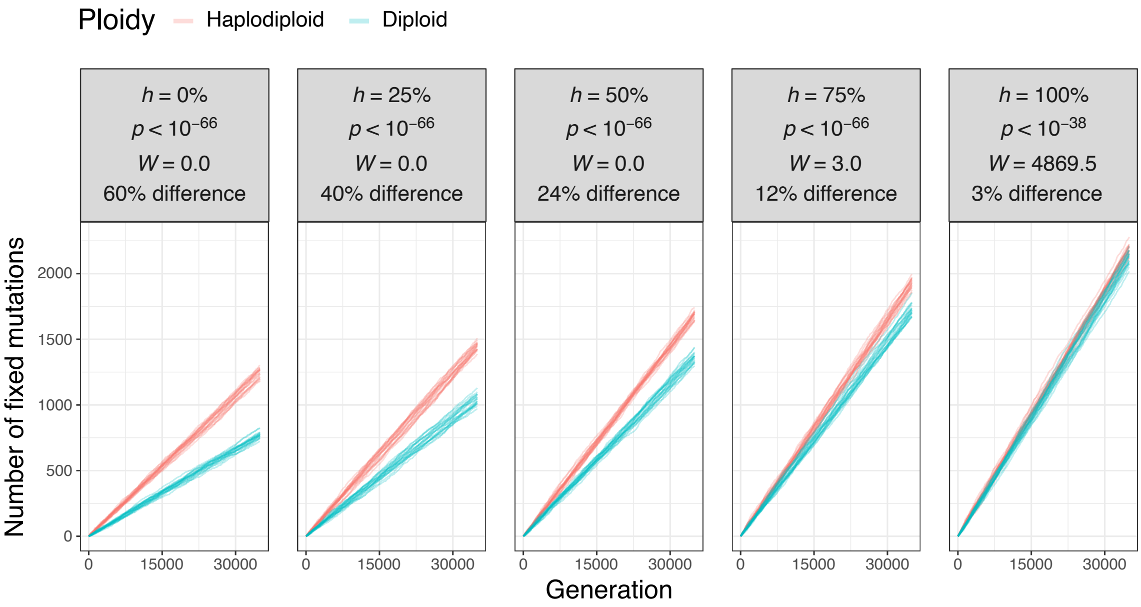

Supplementary figure S1. The effect of haplodiploidy on the fixation rate of advantageous mutations with different dominance coefficients (h). Simulations were run as in fig. 1D, but we increased the number of individuals in haplodiploid populations to $N = 2666$. This increase means that the haplodiploid populations have the same number of chromosomes ($1.5N = 4000.5$) as diploid populations with size $N = 2000$ ($2N = 4000$ chromosomes). On each plot, we also show the average difference in the number of fixed mutations between haplodiploid and diploid simulations after 35,000 generations and a burn-in period of 15,000 generations. As we expected, haplodiploid populations with $h < 1$ had higher fixation rates than diploid populations (Wilcoxon rank sum test, as shown), evidence of a stronger efficacy of selection. At $h = 1$, a modest yet unexpected difference between haplodiploids and diploids may be driven by the interference between positively selected alleles. We expect this interference to be stronger in the haplodiploid populations because of the absence of recombination in males.

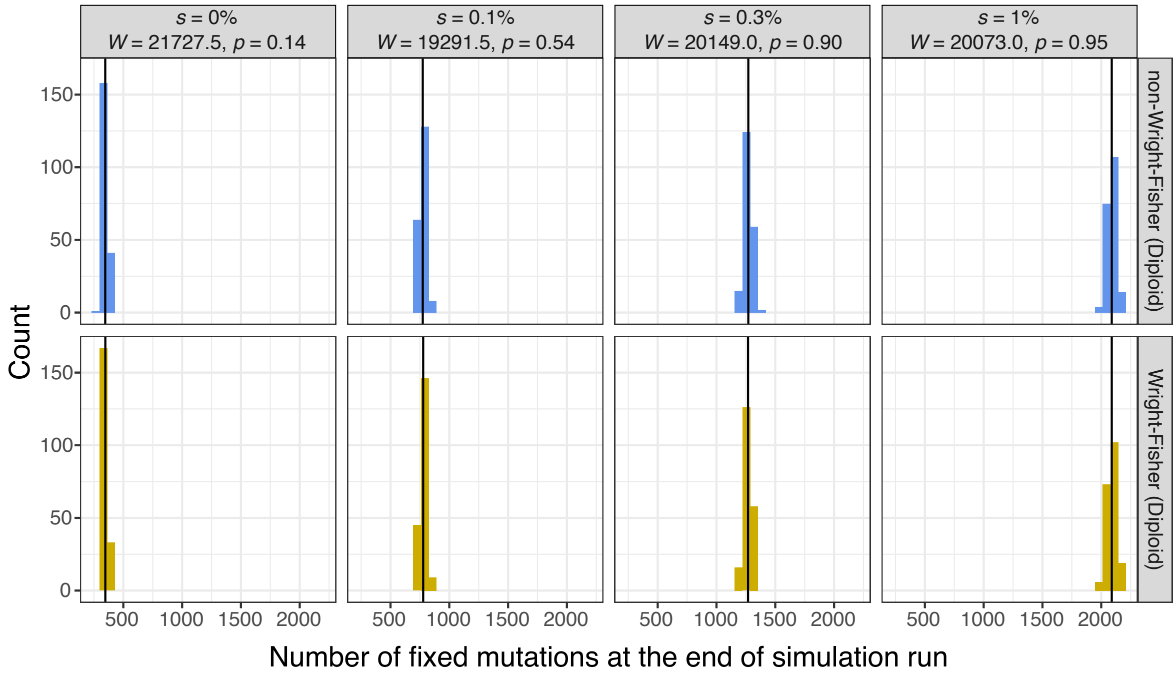

Supplementary figure S2. We compared the non-Wright–Fisher simulations of neutral and advantageous mutations of diploid populations (simulation parameters given in fig. 1) with Wright–Fisher simulations with the same parameters. There was no difference in the number of fixed mutations at the end of the simulation runs between the two types of simulation ($p > 0.05$, Wilcoxon rank-sum tests, as shown). The solid black lines represent the mean number of fixed mutations at the end of the simulation runs for each group.

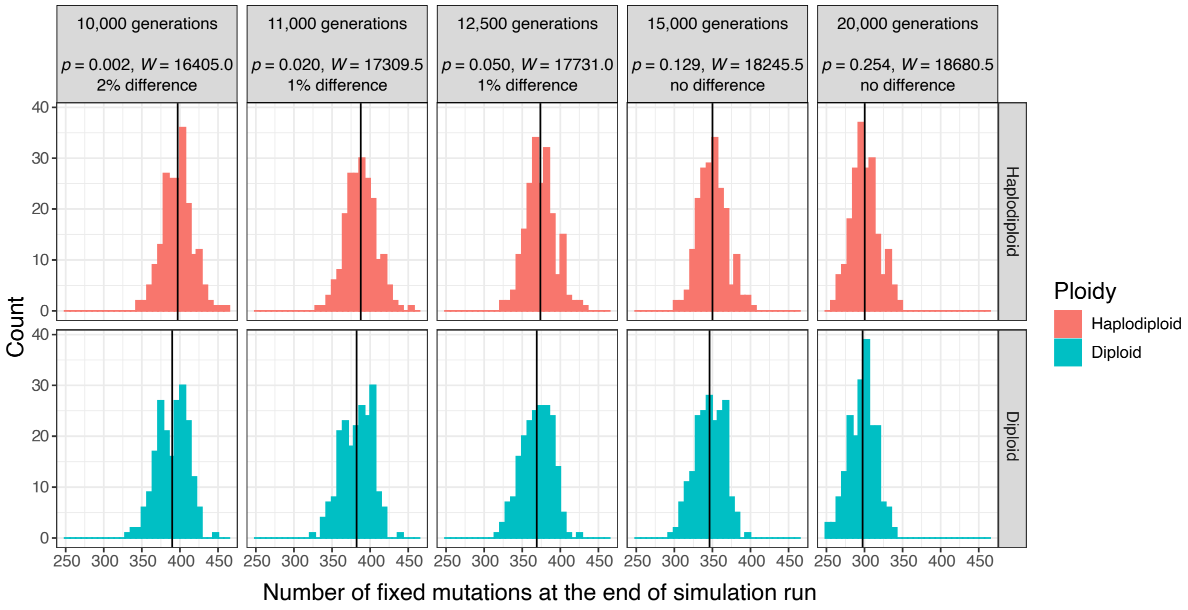

Supplementary figure S3. Choosing appropriate burn-in periods is important. Simulations in which mutations accumulate require a burn-in period of multiple generations before the population reaches mutation–drift balance. Here, we examined the effects of varying burn-in periods out of a total of 50,000 generations. For each tested burn-in period, we performed 200 neutral simulations of haplodiploid populations and 200 neutral simulations of diploid populations ($s = 0$); each population included 1000 male and 1000 female individuals, as in fig. 1A. For each simulation run, we counted the number of mutations that fixed between the end of the burn-in period and the end of the run; solid black lines represent the mean number for each group. Using burn-in periods of 12,500 or fewer generations resulted in a difference between haplodiploid and diploid populations ($p < 0.05$, Wilcoxon rank-sum test, as shown). This artifactual difference is likely to be caused by the lower effective population sizes of haplodiploid populations, which are ¾ those of diploid populations. The smaller effective population sizes lead haplodiploid populations to reach mutation–drift balance earlier, which likely explains the higher number of fixed mutations in these populations.

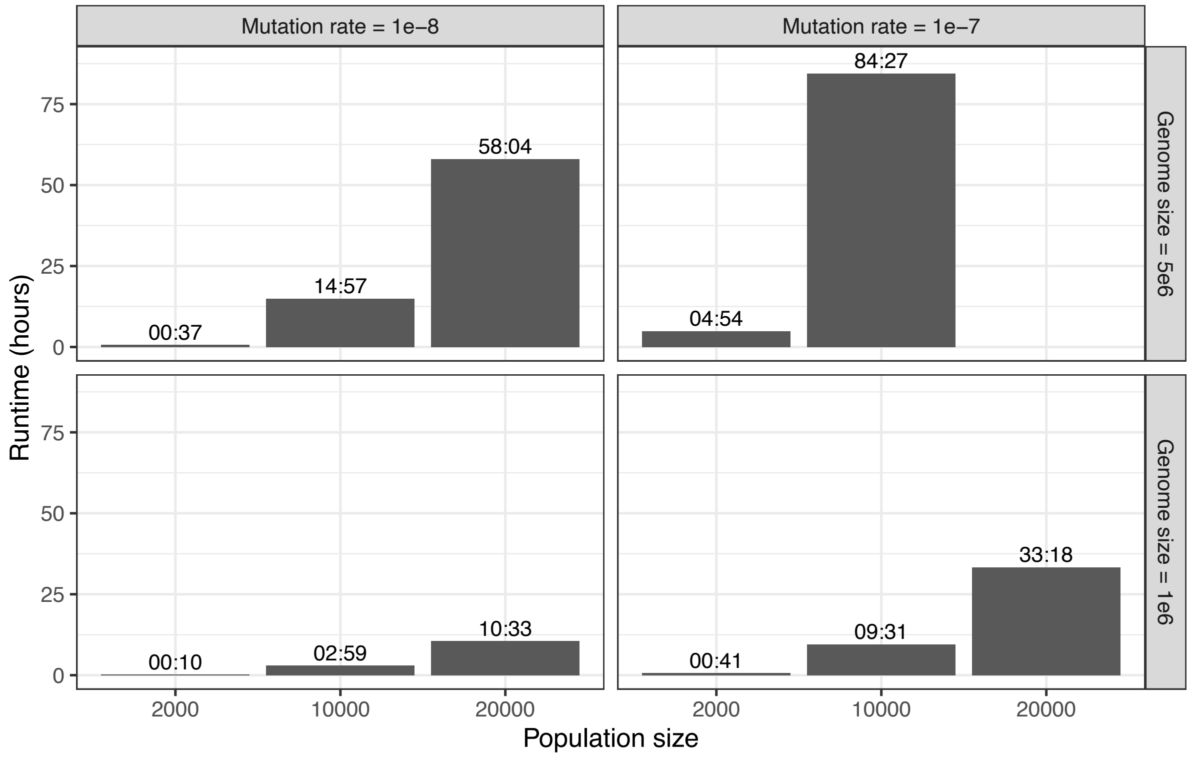

Supplementary figure S4. Runtimes for haplodiploid simulations with 50,000 generations, a recombination rate of 10^{-6}, a 1:1 sex ratio, $s = 0.001$, $h = 0$, and the population size, mutation rate, and genome size shown. The simulations were run using one 2.20GHz core on an Intel Xeon E5-4600 v2 processor on an IBM x3750 M4 (8752) running CentOS Linux release 7.9.2009. The time taken by each simulation is printed over each bar in the format *hours:minutes*.

Supplementary Text

Below, we present the haplodiploidy simulation model in the standard ODD (Overview, Design concepts, Details) format for describing individual-based models (Grimm et al. 2006).

Overview

<u>Purpose</u>

The model is designed to simulate basic haplodiploid inheritance (arrhenotoky), upon which further investigations into the evolutionary genomic dynamics of haplodiploid species can be built. Under haplodiploid inheritance, females are diploid while males are haploid. Females can reproduce asexually to produce haploid male offspring from unfertilized eggs, or sexually with a haploid male to produce diploid female offspring. The model is flexible as it is independent of any particular species or empirical genome sequence. This model provides a starting point for future haplodiploid simulations, since the evolutionary dynamics supported by SLiM include migration, admixture, selective sweeps, complex mating schemes, and continuous-space interactions.

<u>State variables and scales</u>

The implemented model has two hierarchical levels: individual and population. Individuals are characterized by an individual number (an identifier for the particular individual), sex, and age (although generations are currently non-overlapping; every individual lives to age 1). In SLiM, all individuals are "diploid", carrying two copies of a genome of fixed length, which can be populated by mutations with given selection coefficients. For haploid males, the second copy of the genome is kept empty and is designated as a "null" genome (a SLiM concept that facilitates modeling of haploidy and related phenomena). The population is composed of one non-spatial subpopulation of size K with no further variables. Additional individual or population variables could be added to the model.

<u>Process overview and scheduling</u>

SLiM divides events within each generation cycle into discrete phases that utilize 'callbacks': blocks of code which control specific aspects of the simulation. Each generation starts with a `reproduction()` callback, which controls the reproductive events that produce the next generation of offspring. In our model's `reproduction()` callback, individuals reproduce, with a probability proportional to their relative fitness, until K new individuals are produced. The new offspring (aged 0) are added and the old generation (aged 1) is removed in an `early()` callback. Finally, every 100 generations a `late()` callback outputs the generation number and the number of mutations fixed in the simulation thus far.

Design concepts

The effect of fitness is modelled explicitly in the `reproduction()` callback, where an individual's likelihood to be sampled (with replacement) as a parent for the next generation is proportional to its relative fitness. The fitness of an individual is calculated based upon the mutations present in the individual. Females can reproduce sexually or asexually to respectively produce female and male offspring, while males can only reproduce with females to produce female offspring. Outside of reproduction there is no interaction between the individuals in the model as implemented. Mutation and recombination rates (per base position, per generation) are set in the `initialize()` callback at the beginning of simulation. Stochasticity in the model arises from mutation, recombination, and the sampling of parents. To validate the model, we compared the rate of fixation of mutations with different selection coefficients with a parallel model built for diploid populations.

Details

Full code for the model is available as a Supplementary File. Here, we outline the concepts used, in some cases illustrated by code snippets.

<u>Initialization</u>

SLiM models begin with an `initialize()` callback which defines constants and parameterizes the model. In this model's `initialize()` callback the model type is selected to be non-Wright–Fisher so that we can override default reproduction methods. Sex is configured to be modelled explicitly, since otherwise individuals would be hermaphroditic.

We set several parameters that could vary across simulations. A constant representing the population size, `K`, is set to 2000. The mutation rate (per base position, per generation) is set to 10^{-8}. The type of mutations to be modelled is configured with a name (`m1`), a given dominance coefficient, a fixed (`"f"`) distribution of fitness effects, and a given selection coefficient. The type of genomic elements to be simulated is similarly configured with a name (`g1`) and a type of mutations to utilize (`m1`). Importantly, the haploid dominance coefficient "`m1.haploidDominanceCoeff`" of the mutations is set to 1, meaning the selection coefficient of a mutation in a haploid individual is equal to that of a homozygous diploid. A single genomic element of length 10^6 base pairs is set up to use `g1`, representing one chromosome. Finally, the recombination rate for the model (per base position, per generation) is set (10^{-6}).

The last stage of model setup occurs in the first generation of the model, in which `K` individuals are created and added to a new subpopulation, `p1`, with an implicit initial sex ratio of 0.5 (half male, half female).

<u>Input</u>

In this study, we only varied selection and dominance coefficients of the modelled mutations, set in the `initialize()` callback. However, when the model is applied in more species-specific investigations other parameters could be varied or added, and these could change over time or across subpopulations.

Submodels

The haplodiploid mode of inheritance is set up in the `reproduction()` callback. To produce `K` / 2 males for the new generation *t*, `K` / 2 females are sampled from the previous generation *t* – 1:

```
// Identify females
inds = subpop.individuals;
is_female = (inds.sex == "F");
females = inds[is_female];

// Fitness of each female
fit = subpop.cachedFitness(NULL);
FemWeights = fit[is_female];

// Sample females, with replacement, weighted by relative fitness
sampledFemalesHap = sample(females,
                          asInteger(K/2),
                          replace = T,
                          weights = femWeights);
```

This sampling is weighted based on the relative fitness of each female and performed with replacement, so that some females from generation *t* – 1 may produce multiple offspring while others may produce none.

Each female undergoes recombination, with breakpoints generated by SLiM based upon the recombination rate and chromosome length. Note that the haploid male offspring are "diploid" (individuals are always diploid in SLiM), but their second chromosome is kept empty and "null" when they are produced, using the `addRecombinant()` method provided by SLiM:

```
subpop.addRecombinant(strand1 = sampledFemale.genome1,
                      strand2 = sampledFemale.genome2,
                      breaks1 = breaks,
                      strand3 = NULL,
                      strand4 = NULL,
                      breaks2 = NULL,
                      sex = "M");
```

Similarly, `K` / 2 diploid female offspring are produced for generation *t* by the sexual reproduction of `K` / 2 males and `K` / 2 females sampled from the *t* – 1 generation. As with the sampling of females described above, males are sampled weighted by their relative fitness, with replacement. The first chromosome of the diploid females results from the recombination of the mother's genome, and the second chromosome is a copy of the father's haploid genome:

```
subpop.addRecombinant(strand1 = sampledFemale.genome1,
                      strand2 = sampledFemale.genome2,
                      breaks1 = breaks,
                      strand3 = sampledMale.genome1,
                      strand4 = NULL,
                      breaks2 = NULL,
                      sex = "F");
```

To ensure that generations are non-overlapping, we use a `survival()` callback, returning only the new generation of individuals. This also prevents SLiM from using fitness values to govern mortality, which is the default behavior for nonWF models, since we use fitness for mating probabilities instead.

```
survival()
{
	// non-overlapping generations, avoid fitness-based mortality
	return (individual.age == 0);
}
```

After this, a `late()` callback collates measurements about the simulation run every 100 generations, for instance using `sim.substitutions.size()` to count the number of fixed mutations.

Supplementary References

Grimm V, Berger U, Bastiansen F, Eliassen S, Ginot V, Giske J, Goss-Custard J, Grand T, Heinz SK, Huse G et al. (2006). A standard protocol for describing individual-based and agent-based models. *Ecol Model.*, 198:115–126.
